# Antisense Oligonucleotide-Based Splicing Correction in Individuals with Leber Congenital Amaurosis due to Compound Heterozygosity for the c.2991+1655A>G Mutation in CEP290

**DOI:** 10.3390/ijms19030753

**Published:** 2018-03-07

**Authors:** Lonneke Duijkers, L. Ingeborgh van den Born, John Neidhardt, Nathalie M. Bax, Laurence H. M. Pierrache, B. Jeroen Klevering, Rob W. J. Collin, Alejandro Garanto

**Affiliations:** 1 Department of Human Genetics, Radboud University Medical Center, 6525 GA Nijmegen, The Netherlands; lonneke.duijkers@radboudumc.nl (L.D.); alex.garanto@radboudumc.nl (A.G.); 2 The Rotterdam Eye Hospital, 3011 BH Rotterdam, The Netherlands; born@oogziekenhuis.nl (L.I.v.d.B.); L.Pierrache@oogziekenhuis.nl (L.H.M.P.); 3 Human Genetics, Faculty of Medicine and Health Sciences, University of Oldenburg, 26129 Oldenburg, Germany; john.neidhardt@uni-oldenburg.de; 4 Research Center Neurosensory Science, University Oldenburg, 26129 Oldenburg, Germany; 5 Department of Ophthalmology, Radboud University Medical Center, 6525 GA Nijmegen, The Netherlands; nathalie.bax@radboudumc.nl (N.M.B.); jeroen.klevering@radboudumc.nl (B.J.K.); 6 Donders Institute for Brain, Cognition and Behaviour, Radboud University Medical Center, 6525 GA Nijmegen, The Netherlands; 7 Department of Ophthalmology, Erasmus Medical Center, 3015 CE Rotterdam, The Netherlands

**Keywords:** *CEP290*, antisense oligonucleotides, splicing correction, compound heterozygosity, Leber congenital amaurosis

## Abstract

Leber congenital amaurosis (LCA) is a rare inherited retinal disorder affecting approximately 1:50,000 people worldwide. So far, mutations in 25 genes have been associated with LCA, with *CEP290* (encoding the Centrosomal protein of 290 kDa) being the most frequently mutated gene. The most recurrent LCA-causing *CEP290* mutation, c.2991+1655A>G, causes the insertion of a pseudoexon into a variable proportion of *CEP290* transcripts. We previously demonstrated that antisense oligonucleotides (AONs) have a high therapeutic potential for patients homozygously harbouring this mutation, although to date, it is unclear whether rescuing one single allele is enough to restore CEP290 function. Here, we assessed the AON efficacy at RNA, protein and cellular levels in samples that are compound heterozygous for this mutation, together with a protein-truncating mutation in *CEP290*. We demonstrate that AONs can efficiently restore splicing and increase protein levels. However, due to a high variability in ciliation among the patient-derived cell lines, the efficacy of the AONs was more difficult to assess at the cellular level. This observation points towards the importance of the severity of the second allele and possibly other genetic variants present in each individual. Overall, AONs seem to be a promising tool to treat *CEP290*-associated LCA, not only in homozygous but also in compound heterozygous carriers of the c.2991+1655A>G variant.

## 1. Introduction

Leber congenital amaurosis (LCA, OMIM 204000) is the most severe subtype of inherited retinal disease (IRD), and is characterized by a congenital visual impairment, progressive degeneration of photoreceptor cells, sensory nystagmus, and the absence or significant reduction of electroretinographic responses in the first year of life [1]. So far, mutations in 25 different genes have been associated with LCA [2], affecting 1:50,000 individuals [3,4]. Mutations in a gene called the Centrosomal protein of 290 kDa (*CEP290*) are the most frequent cause of LCA in some populations. The protein encoded by this gene is localized at the base of the connecting cilium of the photoreceptors, which is an essential structure to transport proteins from the inner segment, where they are synthesized, to the outer segment, where phototransduction takes place. [5,6]. CEP290 is expressed in almost all cell types of the body, and, in addition to its role in cilium assembly and transport [5,7], is involved in cell division [8]. As a consequence, mutations in this gene can cause a broad spectrum of disorders ranging from purely retinal disease (LCA), to systemic forms such as Senior–Løken, Joubert or Meckel–Gruber syndrome [9,10] which have also renal phenotypes associated.

Despite the existing high genetic heterogeneity in LCA, one particular mutation in *CEP290*, c.2991+1655A>G, accounts for 15% of all LCA cases in some European and North-American populations [9,11,12,13]. The c.2991+1655A>G mutation creates a strong splice donor site that is recognized by the splicing machinery, introducing a 128-bp pseudoexon with a premature stop codon into a proportion of mRNA transcripts [13]. Initially, the ratio of aberrant and correct transcripts observed in fibroblast and lymphoblast cells was 1:1, and therefore the cause of the disease was thought to be a haploinsufficiency [13,14,15]. However, very recently, the use of induced pluripotent stem cell (iPSC)-technology showed that up to 90% of the *CEP290* mRNA transcripts contained the pseudoexon upon differentiation of patient-derived iPSCs to photoreceptor-like cells [16], most probably due to specific retinal splicing factors that are not present in other cell types.

The recurrence of this intronic mutation makes it an interesting target for genetic therapy. Over the last years, we and others have shown that antisense oligonucleotides (AONs) are a powerful therapeutic tool to treat IRDs [14,15,16,17,18,19,20]. These small oligonucleotides can bind complementary to the pre-mRNA, and can modulate pre-mRNA splicing by including or excluding (pseudo)exons, or degrading aberrant transcripts [21]. For the deep-intronic c.2991+1655A>G variant in *CEP290*, AONs have been demonstrated to restore splicing, protein levels and ciliation in patient-derived immortalized lymphoblastoid cells [14], fibroblasts [15,17] and photoreceptor-like cells [16]. In addition, these molecules were able to restore *CEP290* splicing in the retina of a transgenic humanized mouse model, without creating any damage in the retina [17]. All these studies were performed in homozygous patient-derived cells or animal models. In this work, we aimed to assess the efficacy of AONs to correct *CEP290* splicing and the cellular phenotype in compound heterozygous cases, by using patient-derived fibroblast cells where only one *CEP290* allele is represented by the deep-intronic c.2991+1655A>G mutation.

## 2. Results

### 2.1. Clinical Information of the LCA-Affected Individuals

Six patients were previously diagnosed with LCA caused by biallelic *CEP290* mutations. In all six cases, at least one of the two alleles harboured the deep-intronic mutation c.2991+1655A>G. Three patients were homozygous for this variant (LCA1-LCA3), whereas three others (LCA4-LCA6) carried protein-truncating mutations on the other allele. In two patients the p.Gly1890* variant was reported as the second disease-causing allele (LCA4 and LCA6), while in the other (LCA5), the p.Lys1575* mutation was present. In addition, we also obtained a skin biopsy from one of the parents of the homozygous LCA-affected individual LCA3, thus heterozygously carrying the c.2991+1655A>G allele (HET1). All affected individuals were diagnosed with LCA soon after birth and were otherwise healthy. In adulthood, subject LCA6 was found to be infertile due to immotile spermatozoa. This might fit within the spectrum of CEP290-associated non-ocular features, although there is no strong evidence supporting that. A summary of the genetic and clinical information for each individual is shown in Table 1.

### 2.2. AON Efficacy at RNA Level

**Figure 1:**
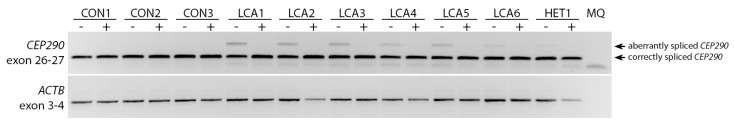
AON rescue at RNA level in all cell lines. RT-PCR analysis of *CEP290* transcripts. The upper band corresponds to the transcript containing the 128-bp pseudoexon. The lower band is the correctly spliced transcript. In all cases that showed pseudoexon inclusion, the splicing was corrected upon AON delivery (+). In the lower panel, actin (*ACTB*) expression is shown as a loading control. Cell lines are indicated with CON (control), LCA (patient) and HET (carrier). MQ corresponds to the negative control of the PCR. (−) means untransfected cells.

Fibroblast cell lines were generated from skin biopsies of all six LCA patients (LCA1-LCA6) and the heterozygous carrier of the c.2991+1655A>G mutation (HET1). In addition, age and gender-matched control cell lines were used. Subsequently, we studied the effect of AON delivery at the RNA, protein and cellular level. Previously, we have identified an AON molecule that is able to efficiently restore *CEP290* pre-mRNA in cell lines derived from patients harbouring the deep-intronic mutation in a homozygous manner [14,17]. In this experiment, we delivered the same AON to all the cell lines at a final concentration of 0.1 µM, and 48 h post-transfection, cells were subjected to RT-PCR analysis. In all seven fibroblast cell lines carrying the deep-intronic variant (LCA1 to LCA6 and HET1), the pseudoexon was detected and efficiently skipped upon AON delivery (Figure 1). In addition, as expected, in the compound heterozygous cell lines (LCA4, LCA5 and LCA6), as well as the one corresponding to the carrier (HET1), a less intense pseudoexon-containing band was detected, due to the fact that the mutation is only present on one of the two alleles. No pseudoexon was detected in any of the control lines (CON1 to CON3) and the delivery of the AON did not appear to alter the normal splicing of *CEP290*.

### 2.3. AON Efficacy at Protein Level

**Figure 2:**
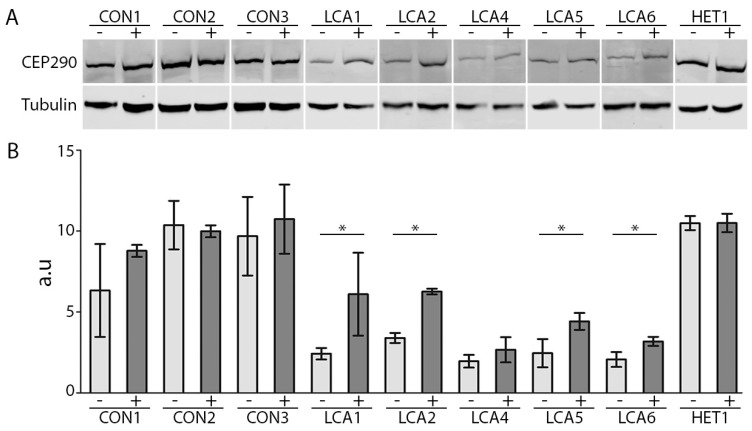
CEP290 protein levels upon AON delivery. (**A**) Immunodetection of CEP290 protein (upper panel) and Tubulin (lower panel) in all controls (CON), LCA-affected patients (LCA) and carrier (HET), in the presence (+) or absence (−) of AON. Tubulin was used as loading control; (**B**) Quantification of the CEP290 protein levels using Fiji software. Bands were quantified twice for each replicate and normalized to Tubulin. Results show the average value for the intensity of the bands and the error bars indicate the standard deviation. The asterisk (*) indicates statistically significant differences (*p*-value < 0.05 using Mann-Whitney test).

We next assessed whether the splicing correction at the RNA level could be translated to an increase in the CEP290 protein levels. For that, an immunodetection of the protein by Western blot was performed. Given the amount of samples, it was not possible to have a direct comparison of all samples on the same gel, and thus we compared untreated versus AON-treated conditions for each cell line. A representative image of the CEP290 protein levels in all cell lines is illustrated in Figure 2A, whereas the quantification of the bands of all replicates is presented in Figure 2B. First, untreated CON and HET1 cell lines showed higher amounts of CEP290 protein compared to the LCA cell lines and no statistically significant differences. Only in one of the replicates for CON1 we observed less CEP290 protein after Tubulin normalization most likely due to some technical limitations. Furthermore, in the homozygous cell lines (LCA1 and LCA2), a clear increase of the protein levels was observed. Of note, after the RT-PCR analysis, the LCA3 cell line was not used for protein analysis and subsequent ciliation studies due to problems in the culturing of this primary cell line. For the compound heterozygous cell lines (LCA4 to LCA6), also an increase of CEP290 protein was observed, however it was less evident compared to the homozygous cell lines, but for LCA5 and LCA6 it was a statistically significant increase. Overall, AON delivery increased the protein levels in all the cell lines carrying the c.2991+1655A>G variant. Moreover, no differences were detected upon AON delivery in control fibroblast cells.

### 2.4. AON Efficacy at Ciliation Level

**Figure 3:**
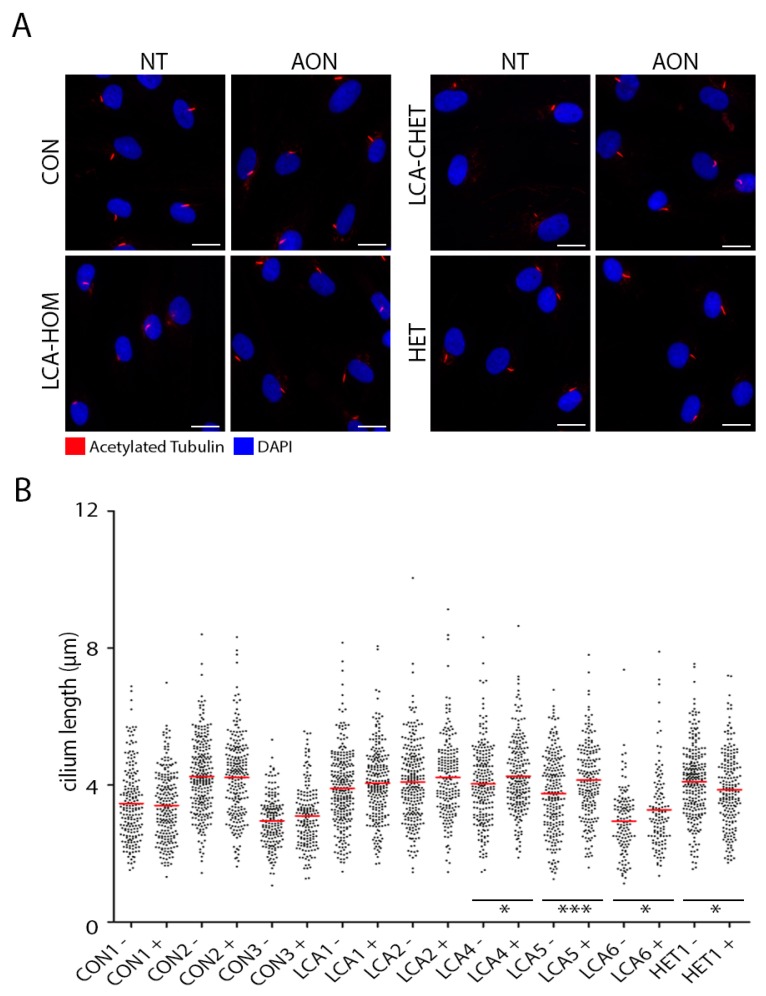
Cilium length assessment in fibroblast cells treated with AONs. (**A**) Representative images of a control (CON), a homozygous LCA (LCA-HOM), a compound heterozygous LCA (LCA-CHET) and carrier (HET) cell line. Nuclei are stained with DAPI (blue) and the axoneme of the cilia using Acetylated Tubulin as a marker (red) in non-treated (NT) and AON-treated (AON) cells. Scale bar represents 20 µm; (**B**) Cilium length analysis of all fibroblast cell lines employed for ciliation studies in the non-treated (−) and AON-treated (+) situation. Control (CON1, CON2 and CON3), homozygous LCA (LCA1 and LCA2), compound heterozygous (LCA4, LCA5 and LCA6) and heterozygous carrier (HET1) cell lines were used in this study. Each dot depicts the length of one cilium and the red line the average length per group. Statistical analysis was performed using Student’s *t*-test. *p*-Values are indicated in the figure. * *p*-value < 0.05 and *** *p*-value < 0.001.

One characteristic of fibroblast cells carrying mutations in *CEP290* is that a cellular phenotype is present. CEP290 is known to function in cilium transport and within the cell cycle, and previously, we and others have observed that in fibroblasts derived from individuals with *CEP290* mutations, these cells show shorter cilia, less ciliation and sometimes even a slower growth speed [15,16,17,22]. AON delivery previously corrected this cellular phenotype in cell lines from patients homozygous for the c.2991+1655A>G mutation [15,16,17]. In this study, we used Acetylated Tubulin as a marker of the axoneme of the cilia (representative images in Figure 3A, images of each condition can be found in Figure S1). Overall, our ciliation studies showed a high variability in the cilium length, even when comparing cells from different control individuals. The cilium length of the control cells was not modified upon AON delivery (Figure 3B). Due to this variability, the AON efficacy was evaluated solely based on the differences between untreated and AON-treated samples for each individual cell line. In LCA1 and LCA2 (homozygous) cell lines, a slight increase was detected, even though, in contrast to our previous study [17], it now was not statistically significant. In contrast, the compound heterozygous cell lines LCA4, LCA5 and LCA6 did show a statistically significant increase in ciliation. In the case of the carrier (HET1) cell line, surprisingly, a significant decrease in ciliation was observed upon AON delivery (Figure 3B).

## 3. Discussion

We here aimed to study whether correcting a single c.2991+1655A>G allele with an AON-based therapeutic approach, would be sufficient to restore the RNA and protein levels, as well as the cilium length, in patient fibroblasts obtained from skin biopsies. Previously we identified an AON molecule that corrected *CEP290* pre-mRNA splicing, increased the CEP290 protein levels and restored a cellular defect in fibroblast cells homozygously carrying the c.2991+1655A>G mutation. These results have recently led to the initiation of a clinical trial (NCT03140969 [23]), assessing the efficacy of AONs in patients harbouring this mutation. However, since the majority of LCA patients with *CEP290* mutations are compound heterozygous for the c.2991+1655A>G variant, it is important to also demonstrate the efficacy of AON-based splice redirection in cells from this group of individuals.

Our study design consisted of three groups of cell lines; the control group, the homozygous group and the compound heterozygous group, together with one carrier cell line. For RNA analysis, we were able to use all cell lines. However, after a very few passages, the *CEP290*-homozygous cell line LCA3 stopped growing and therefore Western blot and ciliation experiments were impossible to perform. One of the reasons could be that when the skin biopsy was received, a contamination was suspected, and the cells were subjected to antifungal treatment with primocin. Any traces of contamination could not be detected afterwards, but this could have had an effect on their normal growth and/or morphology. Another possibility is that these cells carry other variants that may influence the normal cell cycle. In general, we observed that our cell lines were growing at different speeds. On average, the control cell lines, as well as the one derived from the carrier, had a normal speed. The homozygous cell lines, with the exception of the LCA3 case, grew moderately slower than controls. Two of the three cell lines derived from the compound heterozygous patients however grew noticeably slower than the rest of the cell lines. These differences could be related to the severity of the second *CEP290* allele, which represents a protein-truncating mutation. Importantly, we and others have previously described that in c.2991+1655A>G homozygous fibroblast cells, the ratio between correct and aberrantly spliced *CEP290* transcripts on average is ~1:1. This indicates that in a homozygous situation, around 50% of the transcripts are still correct, while in a compound heterozygous situation this is only 25%. Given that CEP290 has been related to cell division, this could explain the evident differences in growth that are observed [8,24,25,26].

The delivery of AONs to cells homozygously harbouring the c.2991+1655A>G mutation was shown to be effective in restoring *CEP290* pre-mRNA splicing, increasing the protein levels and correcting a ciliary defect [14,15,16,17]. However, it was unknown whether correcting one c.2991+1655A>G allele was sufficient. Taken into account that mutations in *CEP290* cause recessive forms of several diseases, we hypothesized that correcting one allele should be enough to restore CEP290 levels and function in the cell. Therefore, we assessed fibroblast cells derived from patients carrying the deep-intronic variant together with a protein-truncating mutation on the second allele. In two cases, the p.Gly1890* variant (LCA4 and LCA6), and in a third one (LCA5), the p.Lys1575* variant were present. Previously, both these mutations were reported to be associated with more severe forms of *CEP290*-associated diseases, i.e., the p.Gly1890* variant with cerebello-ocular-renal syndrome (CORS) and Joubert Syndrome, whilst the p.Lys1575* variant with CORS, Senior-Løken syndrome and Meckel-Grüber syndrome-like (reviewed in [9]). In all cells with the deep-intronic variant, the 128-bp pseudoexon was detected, and it was possible to restore *CEP290* splicing upon AON delivery, independent of the type of mutation present on the second allele. The increase in CEP290 protein was statistically significant in homozygous lines (LCA1 and LCA2), and compound heterozygous lines (LCA5 and LCA6). For LCA4 there was an apparent increase. This rise in CEP290 protein levels also resulted in an increased length of the cilium in all patient-derived cell lines, albeit not always statistically significant. In our previous study, cell lines LCA1 and LCA2 did show a statistically significant increase in ciliation and cilium length following AON treatment [17]. The fact that these cells now only showed a slight increase in ciliation was somehow unexpected. Two explanations for these findings could be (i) the variability of the cilium length that we now observed in our age- and gender matched controls; and (ii) the fact that fibroblasts are primary cells, and therefore they could behave differently after several passages, as we also observed with some control lines in the past. Before, we have used fibroblast cells between passages p4 and p6, whereas now, we used cells between passages p9 and p13. Although we always try to match the passage numbers of our compound heterozygous and control cell lines, this may explain some of the differences we observed between this and our previous study. Nevertheless, our results indicate that it is possible to increase the CEP290 protein expression and the cilium length upon AON delivery when only one of the two alleles is represented by the deep-intronic c.2991+1655A>G variant.

In our study, we also used a cell line of an unaffected heterozygous carrier of the c.2991+1655A>G mutation to assess whether any ciliation defect could be observed in these cells. As expected, the amount of pseudoexon was low, and levels of protein did not show an evident increase after treatment, nor a significant decrease compared to the control cells. Surprisingly, although these cells showed normal levels of ciliation, a statistically significant decrease of the cilium length was detected upon AON administration. Even though both groups followed a Gaussian distribution with a relatively similar cilium length average ± SD of 4.08 µm ± 1.05 for the untreated and 3.88 µm ± 1.09 for the AON-treated group, the medians differed between groups (4.16 and 3.83 µm, respectively). One would assume that one reason could be the amount of cilia counted (*n* = 256 for untreated and *n* = 209 for the AON-treated), however this was taken into account when applying the statistical test. When evaluating the two replicates independently, one showed a statistically significant difference whereas the other did not. When both replicates were combined, these differences became more evident. All samples were treated simultaneously so potential technical problems or alterations due to the reagents could be discarded as this should have then also been observed in other samples. We included age- and gender matched control cell lines, used as lowest cell passages as possible, and tried to compensate the growth speed by seeding more or less cells of each cell line. Nevertheless, the variability between samples and groups was higher than expected.

Parfitt and colleagues recently demonstrated that, by employing induced pluripotent stem cell technology to generate patient-derived optic cups, the amount of aberrantly spliced *CEP290* is much higher in photoreceptor-like cells compared to other cells that were investigated [16]. Therefore, although the generation of these optic cups is a time-consuming and not yet a trivial process, this cellular system would probably be most useful to assess the true ability of AONs to rescue CEP290 protein function in cells from patients with compound heterozygous mutations and illustrates the limitations of the fibroblast system that we have employed here.

Following the use of AONs to rescue the splice defects in *CEP290* associated with the c.2991+1655A>G in humanized transgenic mice, and in cell lines from LCA patients homozygous for this deep-intronic mutation [14,15,16,17], a clinical trial investigating the safety and efficacy of these molecules for the treatment of *CEP290*-associated LCA has now commenced (NCT03140969 [23]). In this study, individuals that are compound heterozygous for the c.2991+1655A>G mutation are also recruited. As these AONs are delivered via intraocular injections, and the main target cells are non-dividing neurons, the duration of a potentially positive effect will mainly depend on the stability of the AON itself. This has been supported by other studies performed in animals using AONs that were delivered to the retina and showed that their effect could be detected after 4–12 weeks [17,27,28,29]. In addition, the first AON drug approved for human administration was Fomivirsen (Vitravene), which was delivered to the retina to treat CMV-retinitis [30,31]. How well LCA patients with compound heterozygous mutations will respond to an AON administration for *CEP290*-associated LCA remains to be established.

In summary, we previously identified an AON molecule that corrected *CEP290* pre-mRNA splicing, increased the CEP290 protein levels and restored a cellular defect in fibroblast cells homozygously carrying the c.2991+1655A>G mutation. Here, we assessed whether the correction of a single allele can also have a potential therapeutic effect. Overall, our results suggest that correcting one allele might be enough to minimize the effects of the pathology, although the severity of the second variant or the presence of other variants in ciliary genes needs to be taken into account.

## 4. Materials and Methods

### 4.1. Study Design

This study was performed using a total of 10 fibroblast cell lines. Groups were divided as follows: three control lines derived from healthy individuals; three c.2991+1655A>G homozygous lines derived from LCA patients; three compound heterozygous lines from LCA patients carrying the c.2991+1655A>G in one allele and a stop mutation in trans (two of them: p.Gly1890* and another p.Lys1575*) and one carrier individual (mother of an affected individual) presenting the deep-intronic variant on one allele. For all experiments all cell lines were processed at the same time and all experiments were performed in duplicate (*n* = 2). The AON efficacy was assessed at RNA level by RT-PCR, at protein level by Western blot and at cellular level by immunocytochemistry (ICC).

### 4.2. Patient-Derived Fibroblast Cells

Our research was conducted according to the tenets of the Declaration of Helsinki. The procedures for obtaining human skin biopsies to establish primary fibroblasts cell lines were approved by the Ethical Committee of the Radboud University Medical Center (Commissie Mensgebonden Onderzoek Arnhem-Nijmegen, file number 2015-1543, permission granted on 28 September 2015). Written informed consent was obtained from all participating individuals.

### 4.3. Cell Culture and AON Transfections

Fibroblast cell lines derived from individuals with *CEP290*-associated LCA and healthy or carrier controls were cultured in Dulbecco’s Modified Eagle Medium (DMEM), supplemented with 20% fetal calf serum (FCS), 1% penicillin-streptomycin and 1% sodium pyruvate at 37 °C and 5% CO_2_. For each analysis, cells were seeded in the corresponding plates. The next day, cells were transfected with AON (0.1 µM final concentration) using FuGENE^®^ HD Transfection Reagent (Promega, Madison, WI, USA) according to the manufacturer’s protocol.

### 4.4. RNA Analysis

Cells were seeded and grown in 12-well plates and transfected with or without AON. Forty-eight-hour post-transfection, cells were harvested and subjected to RNA isolation using Nucleospin RNA isolation kit (Machery Nagel, Düren, Germany) following the manufacturer’s protocol. One microgram of RNA was used for cDNA synthesis using iScript cDNA synthesis kit (Bio-Rad, Hercules, CA, USA). Subsequently, cDNA was diluted by adding 50 µL of H_2_O. PCR was performed with 10 µM of each primer pair, 2 µM of dNTPs, 1.5 mM MgCl_2_, 10% Q-solution (Qiagen, Venlo, The Netherlands), 1 U of *Taq* polymerase (Roche, Penzberg, Germany) and 5 µL of diluted cDNA in a total reaction of 25 µL using the following PCR conditions: 94 °C for 2 min, followed by 35 cycles of 20 s at 94 °C, 20 s at 58 °C and 35 s at 72 °C, with a final extension step of 2 min at 72 °C. Amplicons were resolved in a 2% agarose gel. Actin (*ACTB*) was used as loading control. For *CEP290* the following primers were used: forward 5′-TGCTAAGTACAGGGACATCTTGC-3′ and reverse 5′-AGACTCCACTTGTTCTTTTAAGGAG-3′; for *ACTB*: forward 5′-ACTGGGACGACATGGAGAAG-3′ and reverse 5′-TCTCAGCTGTGGTGGTGAAG-3′.

### 4.5. Western Blot Analysis

Cells were grown in 10-cm dishes and transfected with or without 0.1 µM of AON. Forty-eight hours post-transfection, cells were serum-starved for another 48 h. Subsequently, cells were harvested and homogenized in 130 µL of RIPA buffer (50 mM Tris-HCl pH 7.5, 150 mM NaCl, 1% NP40, 0.1% SDS, 0.5% Sodium deoxycholate, 1 mM EDTA supplemented with protease inhibitors). Total protein was quantified using the BCA kit (Thermo Fisher Scientific, Waltham, MA, USA) as previously described [6,17]. Briefly, for CEP290 immunodetection, ~75 µg of protein lysate was loaded onto a NuPage 3–8% tris-acetate gel (Life Technologies, Carlsbad, CA, USA) and run for approximately 4 h at 100 V. Around 25 µg of the same protein lysates were loaded onto a NuPage 4–12% bis-acrylamide tris-glycine gel (Life Technologies, Carlsbad, CA, USA) for the detection of α-Tubulin. Proteins were transferred to a PVDF membrane (GE Healthcare, Little Chalfont, UK) overnight at 20 V at 4 °C. Blots were blocked in 5% non-fat milk in PBS for 6 h at 4 °C and incubated for 3 days with antibodies against CEP290 (rabbit, 1:750, Novus Biological, Littleton, CO, USA) or α-Tubulin (mouse, 1:2000, Abcam, Cambridge, UK) in 0.5% non-fat milk in PBS at 4 °C. After primary antibody incubation, blots were washed 3 times in PBS with 0.1% Tween-20 for 5 min, incubated with secondary antibody (Goat anti-Rabbit, IRDye 800, 1:20,000 or Goat anti-Mouse, IRDye800, 1:20,000 from Li-Cor Biosciences, Lincoln, NE, USA) for 1 h at room temperature (RT), and washed in PBS with 0.1% Tween-20 for 5 min. Blots were developed using the Odyssey Imaging System (Li-Cor Biosciences, Lincoln, NE, USA). Detected bands were quantified using Fiji Software (1.47v, National Institute of Health, Bethesda, MD, USA) [32]. Band quantification was conducted twice in each replicate and values were normalized to those obtained for Tubulin. Statistical analysis was performed using Mann-Whitney test.

### 4.6. Immunocytochemistry Analysis

Cells were grown on coverslips in 12-well plates and transfected with or without 0.1 µM of AON. Forty-eight hours post-transfection, cells were serum starved for another 48 h. Subsequently, cells were rinsed in 1× PBS, fixed in 2% paraformaldehyde for 20 min at RT, permeabilized in PBS supplemented with 1% Triton X for 5 min at RT and blocked for 30 min in 2% bovine serum albumin in 1× PBS at RT. For the immunostaining, cells were incubated 60 min in blocking solution containing a 1:1000 dilution of an anti-Acetylated α-Tubulin mouse monoclonal antibody (T6793, Sigma Aldrich, St. Louis, MO, USA) at RT. Cells were washed for 3 × 5 min in 1× PBS, incubated for 45 min with the corresponding secondary antibody (1:500 Goat anti-Mouse Alexa fluor 568 (Life Technologies, Carlsbad, CA, USA) diluted in blocking solution. Cells were washed 3 × 5 min in 1× PBS and rinsed in water. Finally, coverslips were mounted in Vectashield with DAPI and imaged on a Zeiss Axio Imager Z1 Fluorescense microscope (Zeiss, Oberkochen, Germany). For each condition, a minimum of 110 ciliated cells were analysed for cilium measurements using Fiji software [32]. Samples were compared using a two-tailed Student’s *t*-test.

## Figures and Tables

**Table 1:** Genetic and clinical information of the individuals.

Individual	Cell Line	Gender	Allele 1 (cDNA)	Allele 1 (Protein)	Allele 2 (cDNA)	Allele 2 (Protein)	Ocular Phenotype	Age at Onset	Extra-Ocular Features
Control 1	CON1	Female	+	+	+	+	Healthy		
Control 2	CON2	Female	+	+	+	+	Healthy		
Control 3	CON3	Male	+	+	+	+	Healthy		
LCA 1	LCA1	Male	c.2991+1655A>G	p.Cys998*	c.2991+1655A>G	p.Cys998*	LCA	birth	none
LCA 2	LCA2	Female	c.2991+1655A>G	p.Cys998*	c.2991+1655A>G	p.Cys998*	LCA	birth	none
LCA 3	LCA3	Female	c.2991+1655A>G	p.Cys998*	c.2991+1655A>G	p.Cys998*	LCA	birth	none
LCA 4	LCA4	Female	c.2991+1655A>G	p.Cys998*	c.5668G>T	p.Gly1890*	LCA/EORP	early childhood	none
LCA 5	LCA5	Male	c.2991+1655A>G	p.Cys998*	c.4723A>T	p.Lys1575*	LCA	birth	none
LCA 6	LCA6	Male	c.2991+1655A>G	p.Cys998*	c.5668G>T	p.Gly1890*	LCA	birth (based on anamnesis)	immotile spermatozoa
Unaffected mother LCA3	HET1	Female	c.2991+1655A>G	p.Cys998*	+	+	Healthy		

CON: control; LCA: Leber congenital amaurosis; HOM: homozygous; COMPHET: compound heterozygous; UNAFF: unaffected; HET: heterozygous; EORP: early-onset retinitis pigmentosa; (+) wild-type.

## Supplementary Materials

Supplementary materials can be found at http://www.mdpi.com/1422-0067/19/3/753/s1.

## Abbreviations

AON	Antisense oligonucleotide
CEP290	Centrosomal protein of 290 kDa
IRD	Inherited retinal dystrophy
LCA	Leber congenital amaurosis
PBS	Phosphate Buffered Saline buffer
PCR	Polymerase chain reaction
RT-PCR	Reverse transcription PCR
RT	Room temperature
