# Supplementary material for: Antisense Oligonucleotide-Based Splicing Correction in Individuals with Leber Congenital Amaurosis due to Compound Heterozygosity for the c.2991+1655A>G Mutation in CEP290
Source: Int J Mol Sci. 2018 Mar 7;19(3):753. doi: 10.3390/ijms19030753 (PMC5877614; doi:10.3390/ijms19030753)
Supplement: Supplementary file 1 [file ijms-19-00753-s001.pdf]

International Journal of *Molecular Sciences*

Antisense Oligonucleotide-Based Splicing Correction in Individuals with Leber Congenital Amaurosis due to Compound Heterozygosity for the c.2991+1655A>G Mutation in CEP290

Lonneke Duijkers [1], L. Ingeborgh van den Born [2], John Neidhardt [3,4], Nathalie M. Bax [5,6], Laurence H.M. Pierrache [2,7], B. Jeroen Klevering [5,6], Rob W.J. Collin [1,6,*] and Alejandro Garanto [1,6]

[1] Department of Human Genetics, Radboud University Medical Center, 6525 GA Nijmegen, The Netherlands; lonneke.duijkers@radboudumc.nl (L.D.); alex.garanto@radboudumc.nl (A.G.)
[2] The Rotterdam Eye Hospital, 3011 BH Rotterdam, The Netherlands; born@oogziekenhuis.nl (L.I.v.d.B.); L.Pierrache@oogziekenhuis.nl (L.H.M.P.)
[3] Human Genetics, Faculty of Medicine and Health Sciences, University of Oldenburg, 26129 Oldenburg, Germany; john.neidhardt@uni-oldenburg.de
[4] Research Center Neurosensory Science, University Oldenburg, 26129 Oldenburg, Germany
[5] Department of Ophthalmology, Radboud University Medical Center, 6525 GA Nijmegen, The Netherlands; nathalie.bax@radboudumc.nl (N.M.B.); jeroen.klevering@radboudumc.nl (B.J.K.)
[6] Donders Institute for Brain, Cognition and Behaviour, Radboud University Medical Center, 6525 GA Nijmegen, The Netherlands
[7] Department of Ophthalmology, Erasmus Medical Center, 3015 CE Rotterdam, The Netherlands
* Correspondence: rob.collin@radboudumc.nl; Tel.: +31-24-3613750; Fax: +31-24-3668752

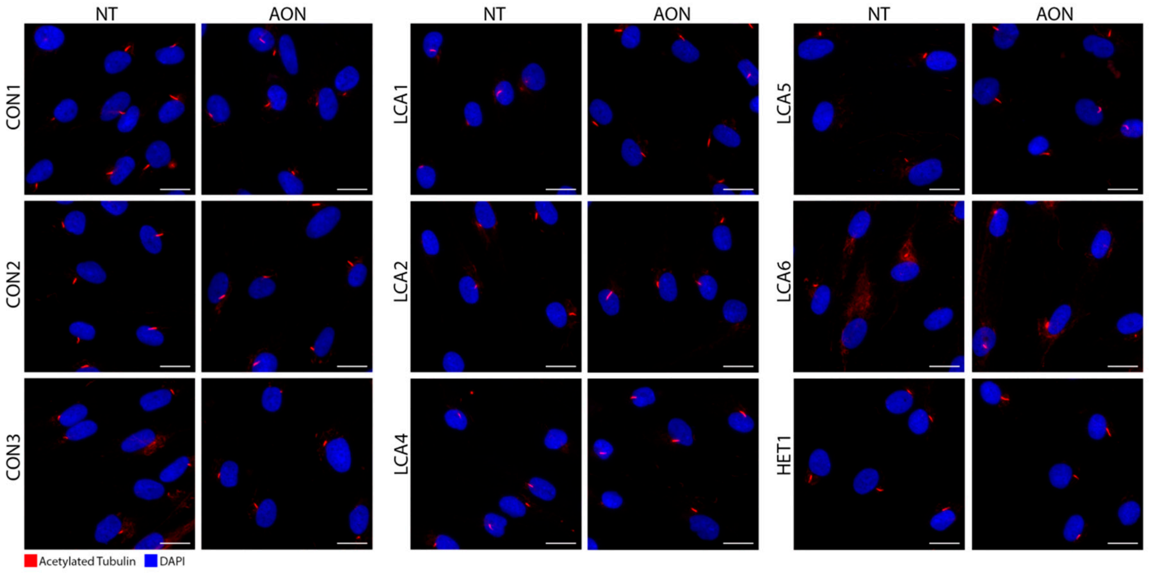

Figure S1. Cilium staining in all fibroblast cell lines. Representative images of each fibroblast cell line used in the ciliation studies. Control (CON), LCA-affected (LCA) and carrier (HET1) cell lines were used for immunocytochemical analysis in the non-treated (NT) and AON-treated (AON) situations. DAPI was used to stain the nucleus (blue) and Acetylated Tubulin for cilium axoneme detection (red). Scale bar represents 20 μm.
